# Risk factors for Baerveldt glaucoma drainage implantation for uveitic glaucoma

**DOI:** 10.1038/s41598-023-29244-1

**Published:** 2023-03-18

**Authors:** Kiyofumi Manako, Eri Takahashi, Junji Saruwatari, Tomoyo Matsumura, Sachi Kojima, Toshihiro Inoue

**Affiliations:** 1 Department of Ophthalmology, Faculty of Life Sciences, Kumamoto University, Kumamoto, Japan; 2 Division of Pharmacology and Therapeutics, Graduate School of Pharmaceutical Sciences, Kumamoto University, Kumamoto, Japan

**Keywords:** Glaucoma, Risk factors, Uveal diseases

## Abstract

Uveitic glaucoma (UG) is sometimes intractable, including intricate interaction between intraocular pressure (IOP) elevation associated with inflammation and side effects of steroids. Based on the Tube Versus Trabeculectomy study in refractory glaucoma results in 2012, tube shunt surgeries have been performed for UG, but few reports have focused on UG. We retrospectively examined the surgical efficacy, complications, and risk factors in 62 eyes with UG that underwent Baerveldt glaucoma drainage device (BGD) implantation at Kumamoto University. The IOPs significantly dropped, and the mean number of glaucoma medications was reduced by more than two. Kaplan‒Meier survival curves were presented under 2 conditions: an IOP reduction of 20% and 6 ≤ IOP ≤ 18 mmHg (criterion A) or 6 ≤ IOP ≤ 15 mmHg (criterion B). In criterion A, the median survival times (MST) were 124 days (complete) and 997 days (qualified). In criterion B, the MST was 129 days (complete) and 867 days (qualified). The Cox hazard proportional model found that the hazard ratio was 0.170 for a history of cataract surgery (95% CI 0.0303–0.950) and 8.669 for systemic immunosuppressive therapy (95% CI 1.810–41.51). BGD implantation is effective for treating UG, but the presence of systemic treatment and the lens status should be considered.

## Introduction

Uveitic glaucoma is a refractory type of glaucoma in which two phenomena need to be controlled: inflammation and IOP elevation^1^. In addition, uveitis sometimes occurs at a young age^2^, so more long-term IOP control is needed in uveitic glaucoma than in the common types of glaucoma that develop in adults to maintain visual function throughout life. Although minimally invasive glaucoma surgery is widely performed even in uveitic glaucoma^3–5^, filtering surgery is thought to be the most useful procedure for uveitic glaucoma^6^. However, the outcomes of trabeculectomy are not always good in uveitic glaucoma, and trabeculectomy has serious complications^7,8^. To improve the problems associated with trabeculectomy, new devices have been developed, such as the Ahmed valve and the Baerveldt glaucoma drainage device (BGD)^9,10^. The Tube Versus Trabeculectomy study showed that tube shunt surgeries have a better long-term outcome and fewer serious complications, such as endophthalmitis, than trabeculectomy^11,12^, so tube shunt surgery has become preferable in uveitic glaucoma. However, most analyses of tube shunt surgery include several types of glaucoma, and studies focusing on uveitic glaucoma alone are not sufficient. In this study, we assessed 62 eyes with uveitic glaucoma that underwent BGD implantation and examined the effectiveness and risk factors for BGD implantation in uveitic glaucoma.

## Results

Sixty-two eyes of 58 Japanese patients with a mean age of 60.6 (SD, 13.9) years, with a range from 26 to 82 years (Table 1), were included, and 54.8% of the eyes had a history of trabeculectomy (twice in 6 eyes). The preoperative mean IOP was 29.62 (SD, 13.9) mmHg, and the number of glaucoma medications was 4.42 (SD, 0.67). Table 2 shows the types and medications for uveitis. Eight patients (3 eyes, sarcoidosis; 2 eyes, Behcet’s disease; 1 eye, ankylosing spondylitis-associated uveitis; 1 eye, Vogt‒Koyanagi‒Harada disease; 1 eye, varicella zoster viral iritis in a patient taking oral steroid therapy for myasthenia gravis) had systemic administration of prednisolone, cyclosporin, or biologics (Table 2). The preoperative inflammation condition of the anterior chamber was grade 0 of aqueous chamber cell grading in 51 eyes, 0.5 in 2 eyes, and 2 in 1 eye. In 8 patients undergoing systemic treatment, grade 0 was observed in 6 eyes and grade 0.5 was observed in 2 eyes.

**Table 1:** Demographic data and glaucoma baseline status in 62 eyes of 58 patients.

Sex [n (%)]	
Female	29 (50.0)
Male	29 (50.0)
Age (y), mean (SD)	60.6 (13.9)
Range, years	26–82
Previous ocular surgery [n (%)]	
Trabeculectomy	34 (54.8)
One time	28 (45.2)
Twice	6 (9.68)
Trabeculotomy ab interno	13 (21.0)
Cataract surgery	34 (54.8)
Vitrectomy	2 (3.23)
Penetrating keratoplasty	1 (1.61)
Preoperative status	
IOP (mmHg), mean (SD)	29.62 (13.9)
Number of medications, mean (SD)	4.42 (0.67)
Surgical eye [n (%)]	
Right	33 (53.2)
Left	29 (46.8)
Place of tube insertion [n (%)]	
Anterior chamber	43 (69.4)
Ciliary sulcus	17 (27.4)
Vitreous cavity	2 (3.23)
Position of plate implantation [n (%)]	
superior temporal	48 (77.4)
Inferior temporal	14 (22.6)
Combination of cataract surgery [n (%)]	8 (12.9)

SD, standard deviation; IOP, intraocular pressure.

**Table 2:** Diagnosis of uveitis and medication for uveitis at baseline.

	n (%)
Diagnosis of uveitis	
Sarcoidosis	8 (12.9)
Behcet’s disease	6 (9.68)
Posner-schlossman syndrome	5 (8.06)
Vogt‒koyanagi‒harada disease	4 (6.45)
Viral anterior uveitis	
Cytomegalovirus	5 (8.06)
Herpes simplex virus	2 (3.23)
Varicella zoster virus	1 (1.61)
HTLV-1-associated uveitis	1 (1.61)
Fuchs Uveitis syndrome	2 (3.23)
HLA-B27-associated uveitis	1 (1.61)
AS-associated uveitis	1 (1.61)
Scleritis-associated uveitis	1 (1.61)
Unidentified	25 (40.3)
Medications for uveitis	
Betamethasone sodium phosphate (eye drops)	26 (41.9)
Fluorometholone (eye drops)	9 (14.5)
Aciclovir (eye ointment) + fluorometholone (eye drops)	1 (1.61)
Subtenon triamcinolone acetonide	3 (4.83)
Prednisolone (oral route)	5 (8.06)
Infliximab + oral prednisolone	1 (1.61)
Cyclosporin + oral prednisolone	1 (1.61)
Secukinumab	1 (1.61)

AS, Ankylosing spondylitis; HTLV-1, human T-cell lymphotrophic virus type 1.

### Surgical outcomes and risk factors

**Figure 1:**
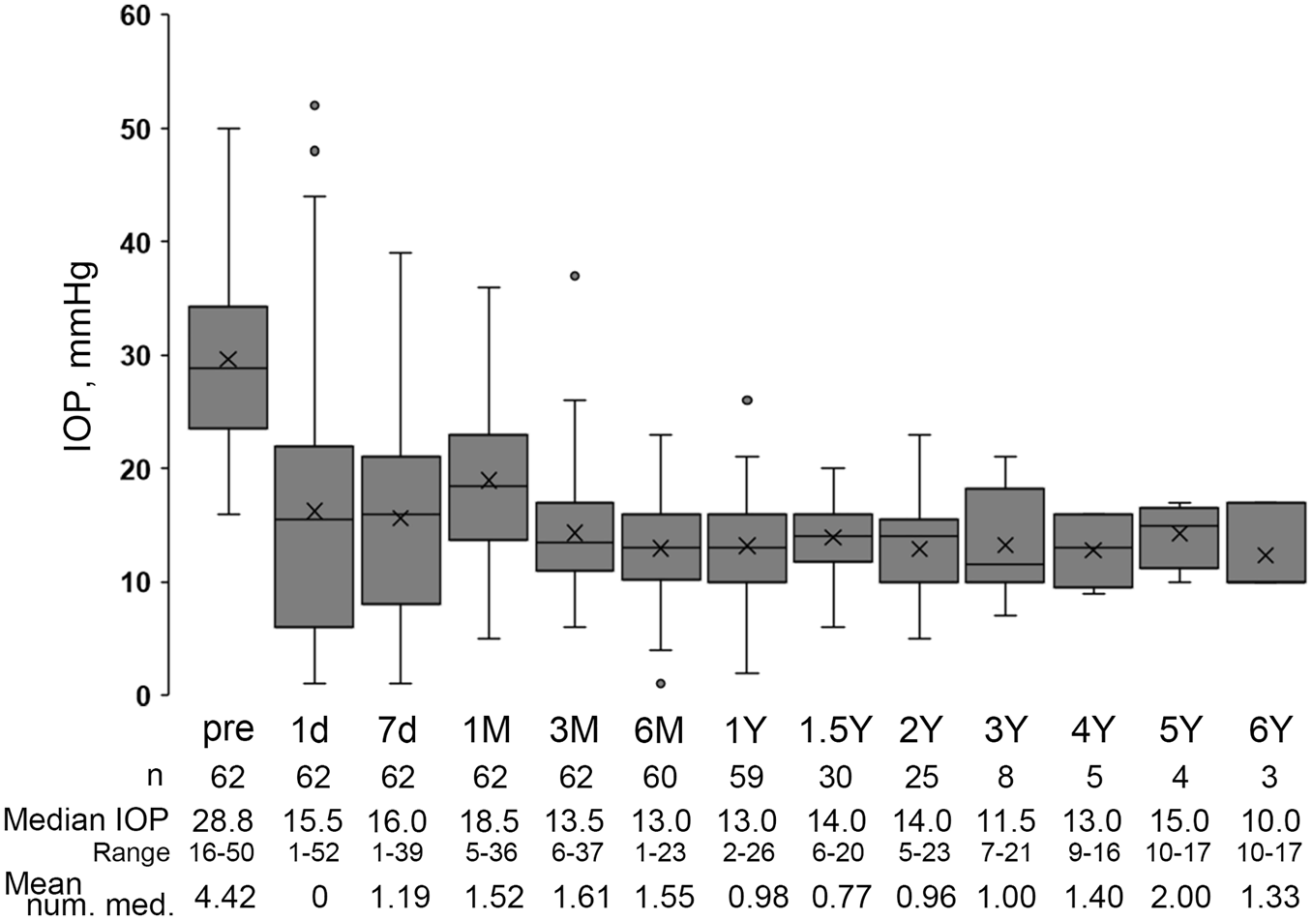
Box plots of the intraocular pressure (IOP) at each time point in all patients. The boxes represent the 25%, median, and 75% IOP, and the cross marks represent the mean IOP.

**Figure 2:**
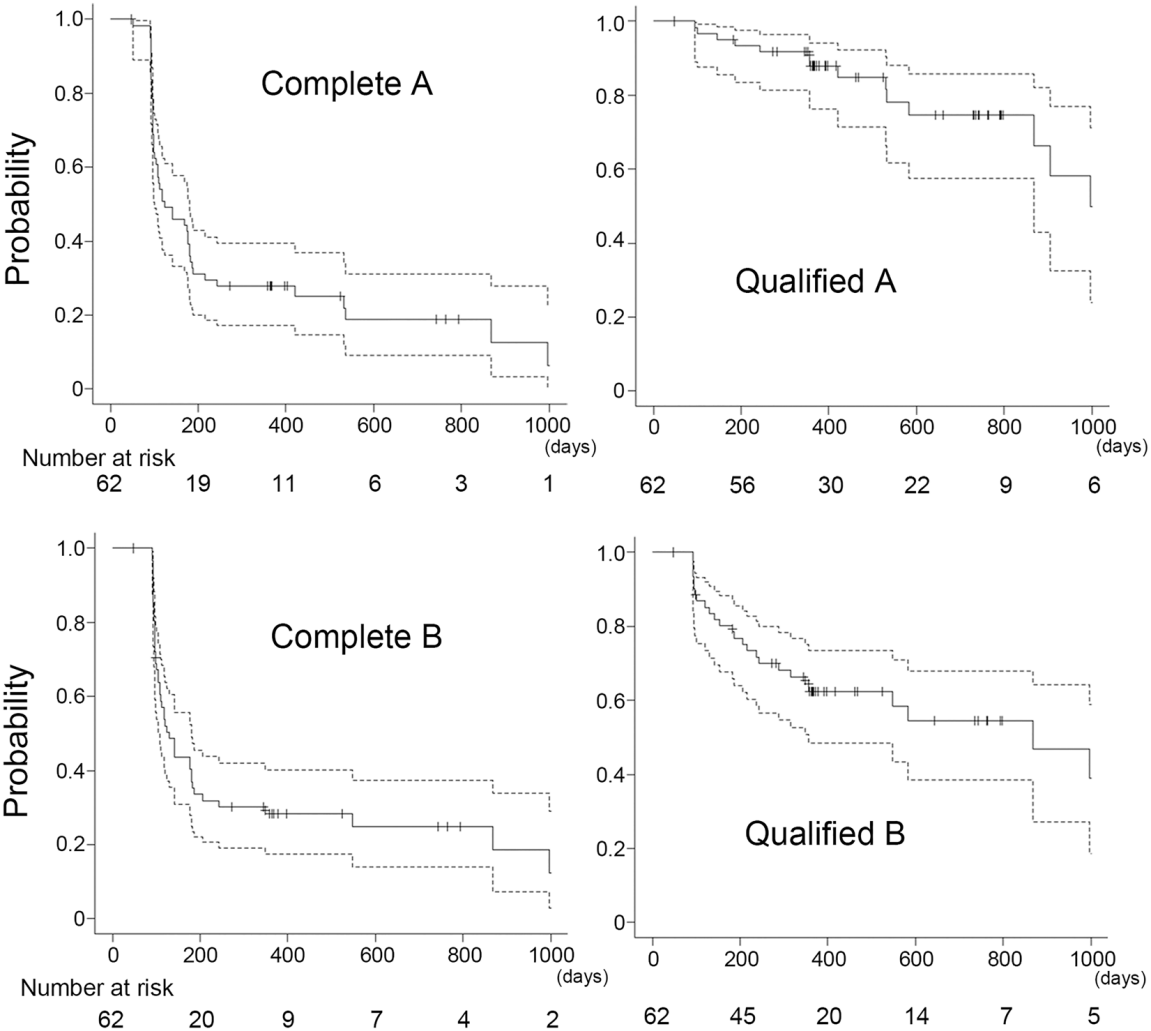
Kaplan‒Meier curve for all patients. Graphs show the curve under criterion A with (Qualified A) or without (Complete A) glaucoma medications and criterion B with (Qualified B) or without (Complete B) glaucoma medications. The dotted lines indicate the 95% confidence intervals.

The time course of IOP levels after surgery is shown in Fig. 1. Although the IOPs varied widely in the early postoperative days (day 1 to month 1 after surgery), the IOPs significantly dropped at all visits after surgery and maintained low levels after 6 months postoperatively (Fig. 1 and Supplementary Table S1). The number of glaucomatous medications also decreased significantly (Fig. 1 and Supplementary Table S1). Figure 2 shows the Kaplan‒Meier survival curves for complete and quantified success of 20% reduction and 6 ≤ IOP ≤ 18 mmHg (A), and 6 ≤ IOP ≤ 15 mmHg (B), respectively. Because it takes almost 6 weeks for the BGD system to work^13^, patients sometimes need glaucoma medications during the early postoperative period (Fig. 1 and Table 3), which may be related to the lack of complete success in the early period. The most common reason for failure in qualified conditions was a higher IOP, and 6 eyes failed due to hypotony. Cox proportional hazard analysis for surgical failure revealed that the hazard ratio (HR) of systemic immunosuppressive therapy was 8.669 [95% confidence interval (CI) 1.810–41.51; *p* = 0.00688] and the HR of previous cataract surgery was 0.170 (95% CI 0.0303–0.950; *p* = 0.0436) in qualified criterion A (Table 4). None of the factors in qualified criterion B were associated with prognosis (Supplementary Table S2).

**Table 3:** Median survival time and 1- and 2-year survival rate.

		MST	95% CI	1-YSR	95% CI	2-YSR	95% CI
Criterion A	Complete	124	101–181	0.279	0.173–0.394	0.188	0.091–0.312
	Qualified	997	867-NA	0.88	0.763–0.941	0.747	0.575–0.858
Criterion B	Complete	129	108–181	0.283	0.176–0.401	0.248	0.139–0.373
	Qualified	867	357–2448	0.624	0.486–0.735	0.546	0.386–0.680

MST, median survival time; YSR, Year survival rate; CI, confidence interval.

**Table 4:** Cox proportional hazard analysis for qualified criterion A for surgical failure.

Factors	HR	95% CI	P value
Age	1.007	0.963–1.053	0.760
Preoperative IOP	1.018	0.936–1.107	0.680
Preoperative steroid drop use	0.299	0.0728–1.231	0.0945
Systemic immunosuppressive therapies	8.669	1.810–41.51	0.00688
Previous cataract surgery	0.170	0.0303–0.950	0.0436
Place of tube insertion	2.731	0.741–10.07	0.131
Position of plate implantation	1.884	0.430–8.255	0.401
Combined with phaco	1.732	0.308–9.750	0.533
Hyphema	4.229	0.827–21.64	0.0834
LSL	< 0.001	0-Inf	0.998

HR, hazard ratio; CI, confidence interval; IOP, intraocular pressure; LSL, laser suturelysis.

### The correlation of cataract surgery and surgical outcome of BGD implantation

**Figure 3:**
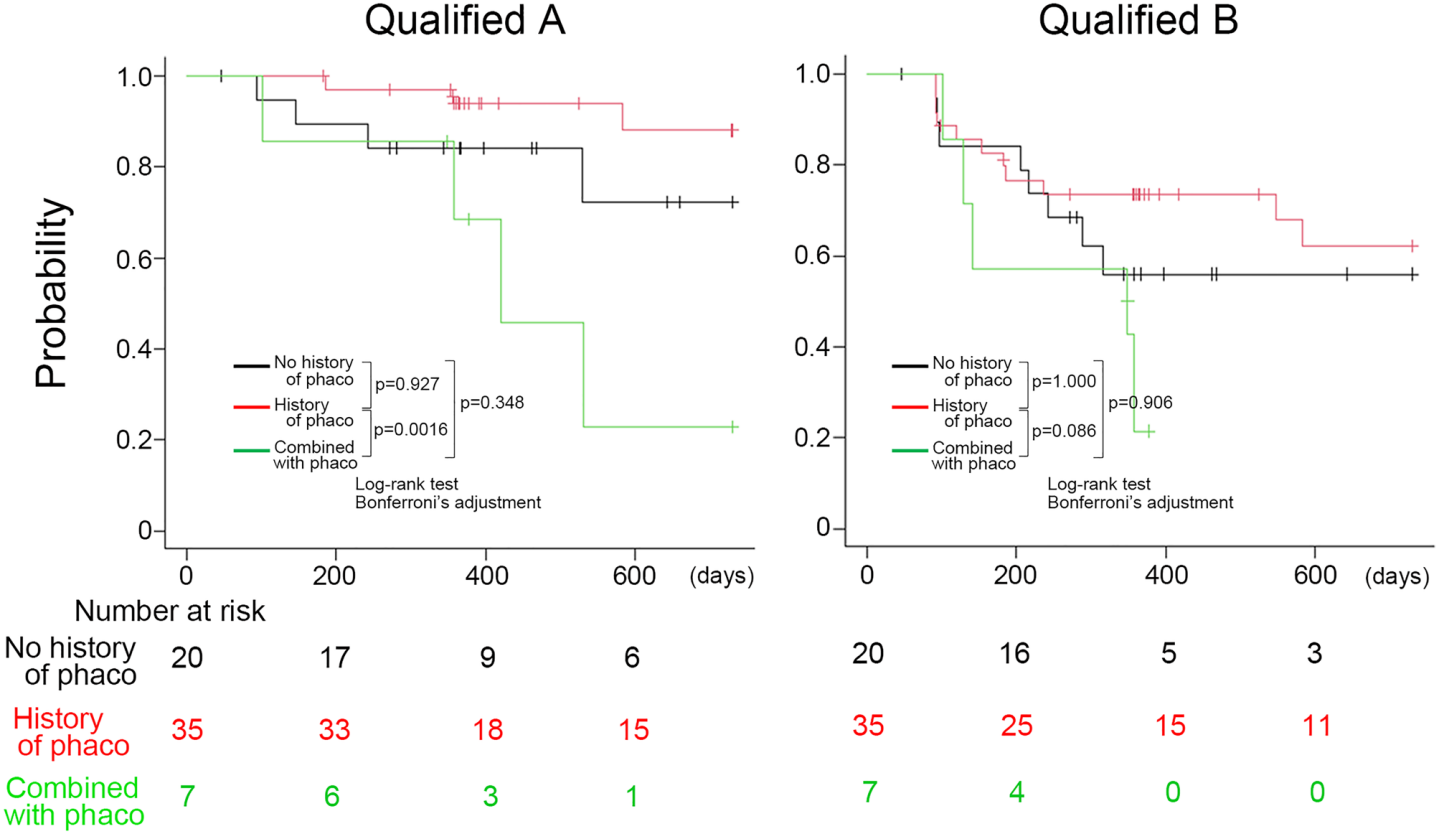
Kaplan‒Meier curve of the eyes with no history of phaco (black line), with a history of phaco (red line), and combined with phaco (green line) under qualified criterion A (Qualified A) and qualified criterion B (Qualified B).

Table 5 shows the patient data categorized by history of cataract surgery. Thirty-five eyes had a history of cataract surgery, 7 eyes underwent BGD implantation combined with cataract surgery, including one eye with intrascleral intraocular lens (IOL) fixation, and 20 eyes had no history of phaco. Ciliary sulcus insertion of the tube was performed in 13 pseudophakic eyes and 4 eyes with BGD implantation combined with phaco (Table 5). We further illustrated the Kaplan‒Meier curve assessing the impact of cataract surgery on the prognosis of BGD implantation (Fig. 3). The eyes with a history of cataract surgery were better than the eyes that underwent BGD implantation combined with phaco in qualified criterion A (*p* = 0.0016), and there were no differences among the three groups in qualified criterion B (Fig. 3).

**Table 5:** Demographic data for three groups classified by history of phaco.

	Previous phaco	Combined with phaco	No history of phaco
n (eyes)	35	7	20
Sex (n, patients)			
Female/Male	20/14	3/4	6/13
Age (y), mean (SD)	64.64 (13.20)	56.49 (18.19)	55.07 (11.67)
Systemic treatment for uveitis	3	2	3
Previous intraocular surgery (n)			
Trabeculectomy	25	1	8
Trabeculotomy ab interno	5	3	5
Vitrectomy	2	0	0
Preoperative status			
IOP (mmHg), mean (SD)	28.71 (7.33)	31.74 (5.49)	30.47 (7.67)
Number of medications, mean (SD)	4.26 (0.7)	4.71 (0.49)	4.60 (0.60)
Surgical eye			
Right/Left	17/18	4/3	12/8
Place of tube insertion (n)			
Anterior chamber	20	3	20
Ciliary sulcus	13	4	0
Vitreous Cavity	2	0	0
Position of plate implantation (n)			
Superior temporal	26	5	17
Inferior temporal	9	2	3

N, number; phaco, phacoemulsification; SD, standard deviation; IOP, intraocular pressure.

### Complications

Table 6 shows the postoperative complications [mean observation period, 668.5 (SD, 572.5) days]. Six eyes had a flat anterior chamber that required injection of viscoelastic material into the anterior chamber, including 3 eyes that underwent additional ligation of the tube. One of the two eyes with acute anterior uveitis was treated with subconjunctival injection of betamethasone valerate. An exposed tube was observed in 2 eyes and was covered with a preserved sclera: one eye had it at 996 days, and the other had it at 189 days after surgery. The latter eye had tube exposure again, and the tube was removed 357 days after surgery. A fibrous membrane encapsulating the bleb around the plate causing IOP elevation was observed in 2 eyes and was removed at 392 days after surgery in one eye and at 817 days after surgery in the other eye. IOL removal and intrascleral IOL fixation were performed for an IOL dislocation 6.4 years after surgery. Eight eyes underwent cataract surgery after BGD implantation. One eye developed central retinal vein occlusion on postoperative day 267 and underwent intravitreal injections of anti-vascular endothelial growth factor to treat macular edema 19 times. Furthermore, the cornea near the insertion of the tube was thinned 2448 days after surgery, resulting in endophthalmitis, and a vitrectomy was performed. The preoperative mean corneal endothelial cell density (CECD) was 1746.5 (SD, 635.0) cells/mm^2^ (n = 61). One eye was unmeasurable due to corneal edema. The postoperative mean CECD was 1734.6 (SD, 645.3) cells/mm^2^ (n = 53). Data from 8 eyes were missing, and 1 eye whose CECD had been unmeasurable preoperatively was unmeasurable postoperatively. The mean observation period at the time of CECD measurement was 505.9 (SD, 1382) days.

**Table 6:** Postoperative complications after BGD implantation surgery.

	n
Flat anterior chamber*	6
Wound dehiscence with leaking of aqueous humor*	3
Hyphema*	8
Vitreous hemorrhage	1
Fibrin formation in anterior chamber*	5
Corneal erosion	2
Tube-corneal endothelium touch*	1
Tube-iris touch*	16
Chorodial detachment*	8
Hypotony maculopathy*	1
Macular edema*	3
Diplopia (temporary)	2
Acute anterior uveitis (recurrence)	2
Herpetic keratitis (recurrence)*	1
Tube exposure*	2
Encapsulating bleb*	2
Dislocation of intraocular lens	1
Central retinal vein occlusion*	1
Endophthalmitis*	1

BGD, Baerveldt glaucoma device. * includes two or more complications in the same eye.

## Discussion

This study is a retrospective study of BGD implantation in eyes with uveitic glaucoma, and survival curves under two criteria were analyzed. In addition, not only ocular conditions but also kinds of treatments against uveitis as prognostic factors were examined by Cox proportional hazard analysis. Our findings showed that the timing of cataract surgery and the presence of systemic immunosuppressive treatments should be taken into consideration as factors associated with prognosis.

In this study, there were significant IOP reductions from 29.6 mmHg to less than 15 mmHg (Fig. 1 and supplementary Table S1), and our 1-year success rate (1-YSR) was 88% under qualified criterion A and 62% under qualified criterion B (Fig. 2 and Table 3). Two previous reports investigated the outcomes of BGD implantation in uveitic glaucoma. Chow et al*.* showed that IOPs dropped from 33 to 12 mmHg at 1 year after surgery, and Tan et al*.* reported that IOPs were reduced from 30.6 mmHg to below 12 mmHg and that the 1-YSRs under the qualified conditions of a 30% reduction of IOP from baseline and 5 mmHg ≤ IOP ≤ 18, 15 mmHg were 87% and 67%, respectively^14,15^. The postoperative mean IOP in this study was slightly higher than that in these previous studies, but the 1-YSR was almost equivalent to the results by Tan et al. In addition, the number of glaucoma eye drop medications decreased from 4.42 ± 0.67 to less than 2 after surgery (Fig. 1 and supplementary Table S1). Taken together, BGD implantation is useful for uveitic glaucoma, as previously reported.

The use of steroids in uveitis management is essential and sometimes causes cataracts as well as steroid-associated IOP elevation, and inflammation itself also causes cataracts and IOP elevation^16^. Therefore, patients with uveitis, even young patients, often require cataract and glaucoma surgery, although the timing of each surgery depends on the severity. On the other hand, the timing of cataract surgery has been implied to influence the prognosis of glaucoma surgery^17,18^. According to reports referring to the effects of phaco on BGD implantation, BGD implantation combined with phaco had worse surgical outcomes than BGD alone^19^, and the prognosis of BGD implantation combined with phaco was worse than BGD alone in pseudophakic eyes in this study (Fig. 3). However, the number of BGD implantations combined with phaco was small in this study, so further research with a large number of patients is needed to verify the effect of the combination procedure on surgical outcomes.

Interestingly, we found that eyes that previously underwent cataract surgery showed a good prognosis (Table 4). One reason for the poor prognosis of the combined procedure is that it may be more invasive for uveitic eyes. On the other hand, the possible reason why a history of cataract surgery has a good impact on the prognosis for BGD implantation is that the anterior chamber is deeper in pseudophakia than in phakic eyes^20^, which may be associated with a lower occurrence of tube-iris touch. In this study, the incidence of iris touch was 45.0% (9/20) in the group with no history of phaco, 12.5% (1/8) in the group with phaco, and 17.1% (6/35) in the group with a history of phaco (Table 6). The contact between the tube and iris may also cause inflammation, and Kwon et al. reported that tube-iris touch is a risk factor with an 8.615 hazard ratio in Ahmed glaucoma valve implantation combined with phaco^21^. The tube should be carefully inserted to avoid touching the iris, and further investigation about the effect of tube-iris touch is needed. In addition, the present study has the limitation that this was a retrospective observational study and that the follow-up period of the patients with no history of phaco was shorter than that in the patients with pseudophakic eyes in this study (Fig. 3). Prospective studies with long-term observations are needed to clarify whether the prognosis of BGD implantation in phakia or pseudophakia is better in uveitic glaucoma. The effect of cataract surgery on IOP after BGD implantation was not evaluated because the patients were dropped from the Kaplan‒Meier survival curve analysis at the time of performing phaco after BGD implantation in this study.

This is the first report showing that patients undergoing systemic treatments have a poor prognosis for BGD implantation (Table 4). Patients who require systemic immunosuppressive treatments may potentially have a high level of inflammation. Therefore, compared to patients not requiring systemic therapy, it is speculated that an inappropriate over response for surgical injuries causes poor prognosis after filtering surgery. Complications associated with fibrosis were observed in 2 eyes of 8 patients who had undergone systemic immunosuppressive treatment: one was fibrous encapsulating, and the other was fibrin formation in the aqueous chamber. In addition, the inflammatory response associated with tumor necrosis factor (TNF)-alpha triggers scleral melting^22^. In this study, the preserved sclera patch melted in one eye of a patient who needed another patch again. There was a patient who received anti-TNF-alpha therapy in this study (Table 2). It is not known which kinds of inflammatory cytokines are involved in postoperative conditions, and it is necessary to examine the impact of various biological agents that have exhibited increased usage in recent decades, including anti-TNF-alpha drugs, on surgical outcomes.

In conclusion, BGD implantation is effective in the long term in uveitic glaucoma. The timing of cataract surgery should be carefully considered, and more attention should be devoted to patients receiving systemic immunosuppressive treatments due to the inflammatory response.

## Methods

### Patients

This retrospective cohort study was approved by the Ethics Committee of Kumamoto University Hospital, and all procedures adhered to the tenets of the Declaration of Helsinki. Uveitic glaucoma was diagnosed by glaucoma specialists based on active inflammation, a previous history of uveitis or ocular findings suggesting a history of inflammation and elevated intraocular pressure. BGD implantation was performed at Kumamoto University from October 2013 to May 2021 in patients with inadequate IOP reduction from glaucoma medications.

### BGD implantation and cataract surgery

After cleaning the lids and conjunctival sac and draping, an eye lid opener was applied. Local anesthesia was injected into the sub tenon, the conjunctiva on the upper (or lower) temporal was incised, and a plate of BGD (Model BG-101-350) was inserted under the super (or inferior) and lateral rectus muscles. When the superior temporal site was intact, the plate was inserted at therein. In eyes with bleb at the superior temporal site or scarring due to previous surgery, the plate was inserted at the inferior temporal site. Prior to plate insertion, the root of the tube was tightly ligated with 8–0 Vicryl in a clean environment. The plate was fixed to the sclera with 8–0 nylon. After trimming the length of the tube, a 23G needle was used to create a tract into the eye, anterior chamber, ciliary sulcus, or vitreous cavity. Then, the tube was inserted into the tract and fixed with 8–0 nylon, and Sherwood slits were made according to the preoperative IOP. The tube was covered with a preserved sclera or scleral flap. The conjunctiva was tightly sutured, betamethasone was injected subconjunctivally, and antibiotic eye ointment was applied at the end of the surgery. Cataract surgery was performed using a standard procedure. Briefly, paracentral corneal incisions were made at 20 G V-lance, and a viscoelastic material was injected followed by continuous curvilinear capsulorhexis. The main corneal incision was made by a slit knife, and hydrodissection was performed. After the nucleus of the lens was removed by phaco and the cortical material was removed by irradiation and aspiration, the capsule was filled with a viscoelastic materials, and an IOL was inserted. In cases of combined procedures, the tube shunt procedure was performed after cataract surgery or after IOL insertion. In the case of BGD implantation after IOL insertion, the viscoelastic material was aspirated and removed after BGD implantation surgery.

### Postoperative management and surgical outcome

Topical antibiotics and steroid eye drops were started the day after surgery and were adjusted according to the postoperative IOP level and inflammation. Laser suture lysis of the 8–0 Vicryl sutures ligating the tube was performed in 4 eyes that had high IOP within 4 to 8 weeks after surgery. The two thresholds for surgical success were defined as follows: criterion A was that the IOP was reduced over 20% from the preoperative IOP and that the postoperative IOP level was ≥ 6 mmHg and ≤ 18 mmHg, and criterion B was that the IOP was reduced over 20% from the preoperative IOP and that the postoperative IOP level was ≥ 6 mmHg and ≤ 15 mmHg on 2 consecutive visits, with (qualified success) or without (complete success) glaucoma medications 2 months after surgery. Failure was defined as an IOP deviating from the definition of criteria, an IOP less than 6 mmHg on 2 consecutive visits, indication for additional glaucoma surgery, or blindness. The patients who had cataract surgery after BGD implantation were excluded from the analysis at the time of phaco. IOP was measured with a Goldmann applanation tonometer, or an iCare tonometer was used when applanation measurement was difficult.

### Statistical analysis

Continuous and categorical variables are presented as the mean ± standard deviation or the median (range) and number (%), respectively. The cumulative incidence of cases that did not result in surgical failure by each criterion was estimated based on the Kaplan–Meier method, and the comparisons were carried out using the generalized log-rank test. The multivariable adjusted HR for surgical failure was also calculated by a Cox proportional hazards model. We incorporated the following covariates into the Cox proportional hazards model: age, preoperative IOP, preoperative steroid eye drop use, systemic immunosuppressive therapies, previous cataract surgery, place of tube insertion, position of plate implantation, combined with phaco, hyphema, and laser suturelysis. A value of *p* < 0.05 was considered statistically significant. To compare the nonnormally distributed continuous variables, the Kruskal‒Wallis test was used with Steel’s multiple comparison to assess the mean IOP or the number of medications before and after surgery. One-way analysis of variance and the chi-squared test were used to compare the other continuous and categorical variables, respectively. All statistical analyses were performed using R software, version 4.0.3 (R Foundation for Statistical Computing).

### Ethics declarations and approval for human experiments

All procedures were in accordance with the Declaration of Helsinki and the ethical standards for the Ethics Committee of Kumamoto University Hospital (Senshin-2347) and its later amendments and ethical standards. Informed consent was omitted by the Ethics Committee of Kumamoto University Hospital because this study was an observational study.

## Supplementary Information

Supplementary Information 1.

Supplementary Information 2.

## Data Availability

The raw data are provided by the corresponding author upon reasonable request.
