# Supplementary material for: Risk factors for Baerveldt glaucoma drainage implantation for uveitic glaucoma
Source: Sci Rep. 2023 Mar 18;13:4473. doi: 10.1038/s41598-023-29244-1 (PMC10024771; doi:10.1038/s41598-023-29244-1)
Supplement: Supplementary file 1 — Supplementary Information 1. [file 41598_2023_29244_MOESM1_ESM.docx]

| **Supplementary Table S1.**  Average of IOP level and numbers of glaucoma medications at each time point. | | | | | |
| --- | --- | --- | --- | --- | --- |
|  | n | IOP (SD), mmHg | p value | Num. Med. (SD) | p value |
| pre | 62 | 29.6 (7.24) | reference | 4.42 (0.67) | reference |
| Day 1 | 62 | 16.2 (11.5) | <0.001 | 0 | <0.001 |
| Week 1 | 62 | 15.6 (8.11) | <0.001 | 1.19 (1.79) | <0.001 |
| Month 1 | 62 | 19.0 (7.63) | <0.001 | 1.52 (1.85) | <0.001 |
| Month 3 | 62 | 14.3 (5.17) | <0.001 | 1.61 (1.58) | <0.001 |
| Month 6 | 60 | 13.0 (4.04) | <0.001 | 1.55 (1.51) | <0.001 |
| Year 1 | 59 | 13.2 (4.02) | <0.001 | 0.98 (1.32) | <0.001 |
| Year 1.5 | 30 | 13.9 (3.20) | <0.001 | 0.77 (1.28) | <0.001 |
| Year 2 | 25 | 12.9 (3.83) | <0.001 | 0.96 (1.46) | <0.001 |
| Year 3 | 8 | 13.3 (4.89) | <0.001 | 1.00 (1.51) | <0.001 |
| Year 4 | 5 | 12.8 (3.27) | 0.0028 | 1.40 (1.95) | 0.0056 |
| Year 5 | 4 | 14.3 (2.99) | 0.011 | 2.00 (2.31) | 0.095 |
| Year 6 | 3 | 12.3 (4.04) | 0.044 | 1.33 (2.31) | 0.10 |
|  |  |  |  |  |  |
| IOP, intraocular pressure; SD, standard deviation; Num, number; Med, medications; p value, Steel's multiple comparison test | | | | | |
