# Supplementary material for: Risk factors for Baerveldt glaucoma drainage implantation for uveitic glaucoma
Source: Sci Rep. 2023 Mar 18;13:4473. doi: 10.1038/s41598-023-29244-1 (PMC10024771; doi:10.1038/s41598-023-29244-1)
Supplement: Supplementary file 2 — Supplementary Information 2. [file 41598_2023_29244_MOESM2_ESM.docx]

| **Supplementary Table S2.** Cox proportional hazard analysis for qualified criterion B for surgical failure. | | | |
| --- | --- | --- | --- |
| Factors | HR | 95% CI | P value |
| Age | 0.999 | 0.968-1.032 | 0.969 |
| Preoperative IOP | 1.000 | 0.941-1.063 | 0.992 |
| Preoperative steroid drop use | 0.717 | 0.314-1.638 | 0.429 |
| Systemic immunosuppressive therapies | 1.464 | 0.451-4.75 | 0.526 |
| History of phaco | 0.563 | 0.190-1.671 | 0.301 |
| Place of plate | 1.392 | 0.501-3.87 | 0.526 |
| Location of tube | 1.462 | 0.637-3.356 | 0.371 |
| Combined with phaco | 2.054 | 0.571-7.394 | 0.271 |
| Hyphema | 2.129 | 0.669-6.771 | 0.201 |
| LSL | 0.826 | 0.0995-6.855 | 0.859 |
|  |  |  |  |
| HR, hazard ratio; CI, confidence interval; IOP, intraocular pressure; LSL, laser suturelysis. | | | |
